# The Effects of Self-Efficacy and Physical Activity Improving Methods on the Quality of Life in Patients with Diabetes: A Systematic Review

**DOI:** 10.1155/2022/2884933

**Published:** 2022-07-27

**Authors:** Sajjad Hamidi, Zahra Gholamnezhad, Narges Kasraie, Amirhossein Sahebkar

**Affiliations:** ^1^Department of Psychiatric Nursing, Iran University of Medical Sciences, Tehran, Iran; ^2^Department of Physiology, Faculty of Medicine, Mashhad University of Medical Sciences, Mashhad, Iran; ^3^Applied Biomedical Research Center, Mashhad University of Medical Sciences, Mashhad, Iran; ^4^Rosenberg School of Optometry, University of the Incarnate Word, San Antonio, Texas, USA; ^5^Biotechnology Research Center, Pharmaceutical Technology Institute, Mashhad University of Medical Sciences, Mashhad, Iran; ^6^Department of Biotechnology, School of Pharmacy, Mashhad University of Medical Sciences, Mashhad, Iran

## Abstract

**Objective:**

The purpose of this systematic review is to study the impact of self-efficacy-improving strategies on physical activity-related glycemic control of diabetes.

**Method:**

This systematic review was conducted based on the PRISMA statement. (“Diabetes” OR “glycemic control”) AND (“exercise” OR “physical activity”) AND “self-efficacy” were searched as keywords in databases including PubMed, Google Scholar, Science Direct, Embase, Cochrane, Web of Science, and Scopus between 2000 and 2019 for relesvant articles.

**Results:**

Two reviewers independently screened articles (*n* = 400), and those meeting eligibility criteria (*n* = 47) were selected for data extraction using a predesigned Excel form and critical appraisal using the “Tool for Quantitative Studies.” Different strategies and health promotion programs such as individual or group face-to-face education and multimedia (video conference, video, phone calls, short message service, and Internet-based education) were used in diabetes self-management education programs. The results of different interventions including motivational interviewing (7 studies), exercise (5 studies), multidimensional self-management programs (25 studies), and electronic education (11 studies) had been evaluated. Interventions with more social support, longer duration, combined educative theory-based, and individual education had better outcomes both in postintervention and in follow-up evaluation.

**Conclusion:**

A combination of traditional and virtual long-lasting self-care promoting (motivating) programs is needed to improve patients' self-efficacy for healthy habits like active lifestyle.

## 1. Introduction

Diabetes mellitus refers to a heterogeneous group of metabolic diseases commonly resulting in high blood glucose levels (hyperglycemia). Diabetic patients are at risk of various complications that decrease their quality of life and increase mortality rates [1]. Premature death and long-term disabling complications make diabetes an expensive illness with a significant economic burden, especially in low- and middle-income countries [2]. Chronic hyperglycemia in diabetic patients leads to vascular damage (macro and micro), which is the main factor for the induction of different cardiovascular, nephropathy, retinopathy, neuropathy, and other complications [3, 4]. Different kinds of synthetic antidiabetic drugs and herbal remedies with high antihyperglycemic activity [1, 5, 6] are in use for patients. However, current medications have not been able to slow down the development of the complications of diabetes [7]. Therefore, self-management and self-care strategies are recommended to improve the quality of life and slow down diabetic complications in patients [8]. Self-care behaviors in diabetic patients mean raising the level of knowledge and information about the complex nature of diabetes and taking actions such as controlling blood glucose, healthy eating, physical activity (PA), and foot care [9]. Although evidence suggests the positive effects of PA on diabetes management, studies have shown a low prevalence of PA in people with diabetes [3]. Improvement of patients' exercise self-efficacy might be influenced by behavior related to PA [10]. Improvement in self-efficacy would facilitate controlling the patient challenges of being physically active. Therefore, patient counselors/educators and other practitioners could beneficially construct efficacy-enhancing programs to improve patients' physical activation [11]. Moreover, recent studies suggest that self-efficacy is one of the most influential factors in the self-care of chronic diseases, especially diabetes [12]. Although several educational interventions based on the theory of self-efficacy have been done to improve self-management and glycemic control in diabetic patients, there is not enough literature review and critical appraisal interpreting the results of those studies. Therefore, a systematic review could help better evaluating the effectiveness of self-efficacy-based educational strategies. The purpose of this systematic review was to examine the impact of self-efficacy-improving strategies on PA-related glycemic control of diabetic patients.

## 2. Method

### 2.1. Protocol

This systematic review was conducted based on the Preferred Reporting Items for Systematic Reviews: the PRISMA Statement 15.

### 2.2. Search Strategy

MESH terms such as (“diabetes” OR “glycemic control”) AND (“exercise” OR “physical activity”) AND (“Self-efficacy”) were searched in various search engines and databases including PubMed, Scopus, Science Direct, Embase, Cochrane, Web of Science, and Google Scholar between 2000 and 2019 for a relevant article. The full text of both the randomized controlled trial and pilot studies written in English was included, and non-English abstracts, original articles, reviews, and grey literature were excluded.

### 2.3. Eligibility Criteria and Study Selection

**Figure 1:**
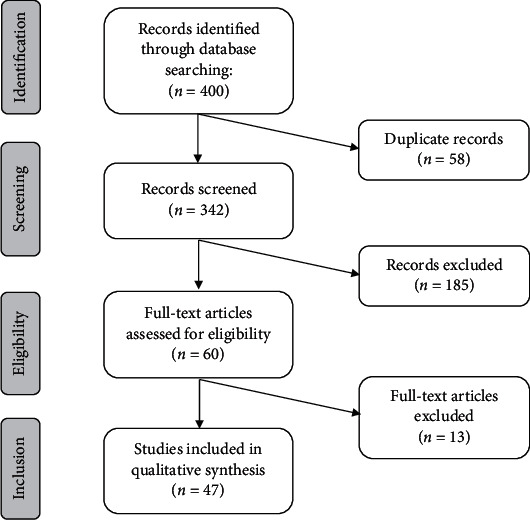
PRISMA flow diagram of the number of studies identified and included in the systematic review.

Four hundred articles were identified in the initial search. All search results were imported to EndNote X8 citation manager, and duplicate studies were removed. Two reviewers independently screened the titles and abstracts of studies to select relevant ones. Disagreements were resolved by consensus. This process resulted in the selection of forty-seven articles for review (Figure 1).

### 2.4. Data Extraction and Quality Assessment

Two reviewers separately collected data from the full texts of the included studies using a predesigned Excel form. Results were compared and double checked by the same reviewers. The data extracted included title, author, year, inclusion/exclusion criteria, design, subjects, strategies of intervention, instruments and measurement, outcome measures, conclusions, and keywords. The methodological quality and validity of each included study were evaluated independently by two reviewers using the “Tool for Quantitative Studies” [13] and Jadad score. Disagreements were resolved by discussion. Studies with no weak rating were defined as strong, with one weak rating as moderate quality, and with more than one weak rating as low (weak) quality. Meta-analysis and outcome measures were not done because of heterogeneity and low quality of the study. Publication bias and statistical analysis were not checked because of low quality and heterogenic studies.

## 3. Results

### 3.1. Self-Efficacy and PA Improvement in Diabetic Patients

Different strategies such as individual or group face-to-face education and multimedia electronic education (education), including video conference, video phone calls, short message service or SMS, and Internet-based education, have been used in diabetes self-management education programs. In addition, motivational interviewing (MI), exercise and education classes, Healthy Eating and Exercise Lifestyle program (HEELP), theory-based group workshops, narrative-based intervention program, peer education program according to health belief model (HBM), home-based exercise program, and other self-management programs had been mentioned in these studies for promoting self-efficacy-related behaviors such as PA in diabetic patients.

### 3.2. Role of MI

The effectiveness of MI in both patients and nurses to develop behavioral changes related to diabetes self-efficacy has been reported. Seven studies used MI as a single educational program or in combination with exercise training to improve self-efficacy and behavioral changes regarding to diabetes self-management. The summarized characteristics of the studies and their quality rating are shown in Table 1. Postintervention evaluation showed improvement of diabetes self-efficacy, active lifestyle, and glycemic control in 5 studies [14–17], and self-efficacy was determined as the main predictor of intention to PA [18]. The motivational intervention was more feasible in women and individuals with a higher educational level [16, 17]. The effect of self-efficacy and intention on exercise performance was mediated by planning strategies [18]. Although these studies reported the positive effect of MI on diabetes self-efficacy as the main predictor of PA intention, there are also negative results [19]. In addition, nurses training for MI of diabetic patients had no significant effect on lifestyle behaviors such as healthy diet, physical activity, and self-efficacy in patients [20].

### 3.3. Role of Health Promotion Programs

Although programs targeting only exercise behavior resulted in patients' active lifestyle behaviors, they did not improve patients' glycemic control. We found five studies using exercise training interventions targeting behavioral changes in diabetes control. Self-monitoring of exercise, home-based resistance training, home-based walking, combination of resistance, and endurance training were the related interventions. Although in some studies, exercise training improved PA self-efficacy [21, 2, 22], and baseline evaluation showed an association between walking ability and self-efficacy; however, in one study, exercise training did not improve self-efficacy-related outcomes of diabetic patients [23]. In addition, glycemic control index (HbA1C) and markers of cardiovascular risk changes were nonsignificantly changed in both intervention and control groups [2]. Low self-efficacy for resistance exercise was the most important predictor of patients' dropout [24], and effective interactions between patients and health care professionals are recommended to encourage patients for behavioral changes and overcoming the barrier to PA [21]. Moreover, it seems that the existence of an underlying disease associated with diabetes has a great impact on study outcomes. It was indicated that individuals without MetS had higher exercise self-efficacy than those with MetS and that home-based exercise programs are beneficial for individuals at risk for diabetes [22].  Table 2 shows a summary of studies using exercise interventions.

Health promotion programs also have been used to evaluate the role of self-efficacy and PA in diabetes management. Different long-term interventions, including Mediterranean Lifestyle Program [25], primary care-based walking program (24 weeks), education programs on exercise-related behavioral changes based on the HBM [26], Healthy Eating and Active Living for Diabetes in Primary care networks (HEALD) program [27], and proactive coping [28], have shown to improve the participants' quality of life (healthy diet, exercise, and stress management) and psychosocial factors (self-efficacy and problem-solving). In some studies, the beneficial effects were sustained even at follow-up evaluation [26], and postprogram contact with patients could improve those outcomes [27]. In another study, with a brief lifestyle self-management program, using follow-up phone calls induced effective lifestyle behavior changes; however, self-efficacy was not increased in the intervention group [29].

Moreover, a combination of theory-based group workshops and walking exercise had a better short-term impact on self-regulation/self-efficacy and PA than online education, but these beneficial effects declined at six-month follow-up [30]. The HEELP program also improved patients' exercise adherence and weight loss; however, the results showed that male gender, self-efficacy, time, and depressive symptoms are independent predictors for exercise duration or BMI change. In addition, lack of motivation and time was the most common exercise barriers at baseline, and there was a negative association between lack of motivation and exercise self-efficacy even after 12-month program [31]. Moreover, other baseline factors, including obesity, coronary heart disease, female gender, self-efficacy, and depressive symptoms, need more attention in designing such programs [32]. Familial factors also might influence the patient's adherence to lifestyle changes. The health stress of patients in the form of higher comorbidity number and specific stress of diabetes in both patients and spouse was inversely correlated with patient adherence to exercise and dietary programs. These effects were mediated by diabetes self-efficacy and depressive symptoms reported by couples [33].

The effectiveness of individual or group self-management improvement methods has been evaluated too. Two studies showed that the patient-centered group education and the structured goal-setting method would lead to better patients' self-management, and the effect of time-by-treatment interaction might partially be mediated via the development of self-efficacy ^38^ [34]. ,In another RCT study, individualized education (IE) had better outcomes compared to group education [35], and long-term evaluation indicated behavioral and psychological improvement in IE; however, this intervention did not show sustained improvement in HbA1c, nutrition, and PA scores [36]. Face-to-face education program targeting self-efficacy on self-care skills resulted in better patient glycemic control, diet, medication adherence, and PA improvement [37, 38]. Face-to-face and five telephone lifestyle counseling sessions on changing the psychosocial determinants of PA and diet also improved patients' self-efficacy and reduced barriers to active lifestyle such as lack of motivation and energy in the intervention group compared to the control [39]. In a RCT study, a program for improvement of worksite lifestyle in prediabetes employees resulted in better behavioral outcomes such as PA and diet self-efficacy and goal commitment [40].

A self-management coaching program on lifestyle changes had more impact on people with lower self-efficacy [41] and social cognitive (self-efficacy) and self-regulatory (illness beliefs) theory-based intervention programs caused a significant improvement in self-efficacy for exercise [42]. Data showed that illness beliefs play an essential role in patients' quality of life, while self-efficacy had a crucial role in self-management behaviors diabetes care providers [42].

Other interventions such as a narrative-based intervention program [43], Spanish Diabetes Self-Management Program [44, 45], nurse-managed health promotion program [46], and prevention program on self-efficacy [47] improved self-management and self-efficacy controlling the disease, although an independent association between social-environmental, problem-solving, and self-efficacy factors with exercise and diet-related behaviors has been reported. However, the development of these psychosocial and social-environmental factors could improve diabetes self-management [48]. Improvement of the knowledge about the importance of exercise and self-efficacy in diabetes care providers leads to better performance in patients' exercise learning [49]. Among diabetic patient counselors/educators, factors such as “time allotted for delivering diabetes self-management/support visits” and “inability to engage patients in physical activity” were identified as practice and challenging barriers. To improve physical self-efficacy in patients, educators challenging problems need attention [50].  Table 3 shows a summary of studies using health promotion programs.

### 3.4. Role of Multimedia and Education

The modulatory effect of self-efficacy on increasing self-care behaviors of diabetic patients was evaluated using different multimedia- and education-based interventions. Education of diabetic patients using a multimedia- (CD-) based health promotion model might improve subjects' beliefs about PA and increase their adherence to exercise [52]. Brief proactive telephone “coaching” interventions also increased patient adherence to exercise and a healthy diet and reduced medical complications and depression. Results showed the beneficial impact of awareness of self-care goals, self-efficacy, and reinforcement on foot inspection, psychological symptoms (depression), and PA [53]. Diabetes educators could apply integrative health coaching for the improvement of patient self-efficacy [54].

In a tailored Internet-based intervention, patients with the highest self-efficacy had better outcomes; therefore, self-efficacy may play a moderator role in intervention outcome and should be considered in tailoring educational intervention for diabetes [55]. In addition, online program (algorithm-driven) for diabetes prevention and improvement of diabetes self-management, self-efficacy and satisfaction, can result in promoting PA behavior [56]. However, although online education was shown to improve HbA1C, exercise, patient activation, and self-efficacy, but reinforcement or follow-up had no beneficial effect [57]. Smartphone communication also increased the patients' self-efficacy compared to the control group [58]. However, multimedia education had a better effect compared with short message service (SMS)-based model on patients' self-efficacy and their belief about PA behavior [59].

There are also studies with negative results. Computer-based multimedia program in the waiting administration room of diabetic patients had no significant difference in glycemic control, self-efficacy, and other self-management behavior related to diabetes [60]. Moreover, one-month mobile-based intervention pilot study did not show any significant changes in patients' glycemic control, self-efficacy about food intake, PA, and body mass index [61].  Table 4 shows a summary of studies using multimedia and education.

## 4. Discussion

Motivation had been introduced as a pivotal factor for the improvement of lifestyle, especially in behavioral and psychological aspects, because it increases the learner's effort and desire for a certain change and purpose [62]. MI as a single strategy or in combination with other programs (exercise, healthy diet) has been performed in seven studies. The duration of the studies (RCT and pilot) was between 2 and 12 months, and their population size was 12-152. Although four studies reported improved self-efficacy and PA, one study showed no change in PA. Encouragement of the patient in achieving the goals of diabetes self-management shall be considered as a cost-benefit method in education even with no change in HbA1c and PA.

Our search resulted in five studies with a population of 48-145 which evaluated the effect of endurance or resistance exercise behavior on health-related behaviors (e.g., exercise self-efficacy) and/or glycemic control. According to them, low efficacy of exercise has been proposed as a significant predictor of patients dropping out, and just one study reported the improvement in patients' self-efficacy but had no effect on glycemic control or other diabetes complications. The goal of active life is to improve metabolic status and reduce the complications of diabetes. Moreover, most people with diabetes or metabolic disease have low self-efficacy, quality of life, and knowledge/belief about their illness [22]. Therefore, multifaceted health promotion programs should be applied to cover all psychological and behavioral aspects of lifestyle and induce effective changes in patients' beliefs. A systematic review about lifestyle intervention in diabetic patients suggested future interventions targeting health promotion behaviors with emphasize on problem-solving skills and self-efficacy; but there was no recommendation for the best strategies [8].

A multiconceptual basis education strategy (a combination of goal system and social, cognitive, and ecological theory) was associated with better outcomes. In this survey, twenty-five studies with a sample size of 62-550 and a duration of 3 weeks to one year had been assessed, which used multidimensional self-management programs with both individual/group face-to-face sessions and multimedia training. According to the findings, interventions with more social support, longer duration, combined educative theory-based, and individual education had a better outcome after intervention and follow-up evaluation. In addition, the improvement of the knowledge and self-efficacy of diabetes care providers has not resulted in an increase of exercise self-efficacy in patients with diabetes.

Recently, researchers have been interested in educational technologies such as online and virtual training, multimedia, and smartphone health informative applications to provide more effective health promotion interventions. We found eleven trials with sample sizes of 56-760 and duration trials of 1-24 months. Although patients' feedback about participation in e-education was positive, however, as similar as face-to-face methods, two studies with a small study population and short duration showed no change in outcome. It seems that poor baseline motivation, self-efficacy, and depressive symptoms need more attention in designing such programs [32]. In literature review 2, systematic reviews and meta-analysis evaluated the effectiveness of 15 and 16 studies based on “peer support on self-efficacy” and “self-efficacy-focused education.” Although peer support did not induce any significant change in self-efficacy and quality of life, however, intervention with long duration (>6 months) had a better effect on patients improvement of quality of life [63] which is in line with findings of the present study. Meta-analysis of 10 selected studies from 16 interventions showed the beneficial impact of “self-efficacy-focused education” on glycemic control and quality of life in a patient with type 2 diabetes, but the lack of high-quality rating studies with good emotion/physiological strategies and complete outcome assessment makes it difficult to choose the best strategies [64].

In this systematic review, different methodological approaches for the development of self-efficacy and physical activity in diabetic patients had been summarized and discussed to facilitate the patients' and researchers' access to available studies and their outcomes. This review tried to show the importance of self-management programs in controlling diabetes and emphasized the need for designing most effective methods in improving self-efficacy-focused education. However, this systematic review has several limitations worth mentioning. First, most of the studies were rated as moderate and weak quality with performance bias and detection bias (i.e., lack of double blind, standard randomization, and description of withdrawal). Secondly, the studies were heterogeneous. Study's characteristics, such as population (i.e., number, sex, race, age, education, and concomitant disease), inclusion and exclusion criteria, duration, design (i.e., RCT, prospective observational study, and cross-sectional study), and self-management improving methods were heterogeneous. The lack of enough studies with RCT design and limited number of participants in them make meta-analysis impossible. Moreover, the outcomes of the studies, especially with respect to behavioral outcomes, were also heterogonous because different scales had been employed for self-efficacy and self-management assessment, and different primary and secondary outcomes had been reported. Finally, we evaluated the available English reports (full text) of studies; therefore, potentially relevant reports in other language might have been missed. Taken together, the most important limitation of this study was insufficient high-quality RCTs with enough sample size, long-term education, and follow-up periods, which applied physiological/emotion arousal educational strategies and employed complete outcome assessments with standard scale. Therefore, we could not evaluate the validity and reliability of the instruments and the related outcomes. Regarding those limitations, it is difficult and even impossible to perform a meta-analysis study and combine the findings for achieving descriptive and practical conclusions. Therefore, the impact of self-efficacy-focused education programs including practicing the self-efficacy improvement skills, peer models, goal setting, positive feedback, and health provider persuasion methods on diabetes management is still under question.

## 5. Conclusion

A combination of traditional and virtual long-lasting self-care promoting (motivating) programs with good emotion/physiological strategies is needed to improve patients' self-efficacy for healthy habits like an active lifestyle. Family and social support play an essential role in establishing healthy behavioral changes in diabetic patients. Future high-quality RCT studies with larger sample size, self-efficacy-focused education-based strategies, long duration and follow-up, and standard outcome assessments are needed to evaluate the effectiveness of self-management strategies.

## Figures and Tables

**Table 1:** Characteristics of studies using motivational interviewing in both patients and nurses.

Author, reference	Study design/subjects	Intervention	Instruments/measurements	Outcomes/finding	Quality rating
Swoboda et al. [14]	Randomized pretest-posttest controlled study; adults with ype 2 diabetes (*n* = 54)	One in-person motivational interviewing and decision support session followed by 7 biweekly telephone coaching calls (16 weeks)	The 8-item diabetes self-efficacy scale measures at baseline and upon completion of the 16-week intervention	↑ In diet quality, diabetes self-efficacy, and diabetes empowerment, and a ↓ in diabetes distress and depressive symptoms	Weak
Galle et al. [16]	Pilot quasiexperiment; 81 overweight type 2 diabetic patients	Nine-month multidisciplinary community-based educational (motivational, nutritional, and exercise) program	Satisfaction, worry, and embarrassment regarding their condition, together with disease-related behaviors and propensity towards physical activity	↑ Self-management and patient glycemic control, especially women and individuals with a higher educational level	Moderate
Soderlund et al. [17]	Latinas (*n* = 12) at risk/diagnosed with type 2 diabetes mellitus	Two one-to-one MI and PA sessions were conducted over 2 months	PA, PA stage of change	↑ Adherence to PA in type 2 diabetic women	Weak
Pinidiyapathirage et al. [18]	Quasiexperiment; women with gestational diabetes mellitus (*n* = 152)	Participate in a survey 6–36 months postdelivery	Postal and telephone surveys that collected socio-cognitive and physical activity data	Identified predictors of physical activity among women with previous GDM	Moderate
Locke et al. [15]	Pilot study, type 2 diabetes (T2D) randomized to HIIT (*n* = 15) or MICT (*n* = 17)	Two-week 10 exercise sessions accompanied by a brief 10-minute counselling intervention	Self-efficacy and moderate to vigorous physical activity (MVPA) were measured at baseline. Postintervention and 24 weeks following a brief counselling intervention combined with either HIIT or MICT	Both groups increased in their self-regulatory and task self-efficacy postintervention, but both groups demonstrated similar decline at 24 weeks	Weak
Gillison et al. [19]	Pilot quasicontrol trial study; people at high risk of diabetes or heart disease (*n* = 108)	Eight-month group-based sessions designed to promote motivation, social support, self-regulation, and understanding of the behavior change process	Behavioral and physical activity changes were assessed by questionnaire at baseline, 4 and 12 months	↑ Self-efficacy and motivational factors related to dietary behaviors, it did not change the physical activity	Weak
Heinrich et al. [20]	Randomized controlled study; thirty-three nurses and 584 patients participated	Nurses training for motivational interviewing (MI) of diabetic patients aimed to develop behavioral	Self-administered, written questionnaire with mainly validated scales on self-management behaviors at baseline, after 12 months and after 24 months	MI had no significant effect on lifestyle behavior such as healthy diet, physical activity, and self-efficacy in patients	Moderate

**Table 2:** Characteristics of studies using exercise-based interventions.

Author, reference	Study design/subjects	Intervention	Instruments/measurements	Outcomes/finding	Quality rating
Gleeson-Kreig [21]	Randomized pretest-posttest controlled study; adults with type 2 diabetes (*n* = 58)	Six weeks of self-monitoring of exercise	The self-efficacy and physical activity index scale measured at baseline and upon completion of the 6-week intervention	↑ PA self-efficacy after daily activity recording	Weak
Plotnikoff et al. [2]	Randomized control trial; type 2 diabetic obese patients (*n* = 48)	Home-based resistance training (3 days/weeks for 16 weeks)	Muscle strength, glycemic control, and social cognitions (self-efficacy and intention) to perform exercise evaluated at baseline and postintervention	↑ Body strength, intention, and exercise self-efficacy	Moderate
Collins et al. [23]	Randomized control trial; diabetic patients (*n* = 145) with peripheral arterial disease	Home-based walking intervention for 6 months	Self-efficacy for managing chronic disease scale, mean maximal treadmill walking distance	Baseline evaluation showed an association between walking ability and self-efficacy, but intervention did not changed self-efficacy-related outcomes	Moderate
Nam et al. [24]	Randomized control trial; adult patients with type 2 diabetes (*n* = 140)	Exercise (combination of resistance and endurance training for 6-month, 3 times per week)	Mood states questionnaire, health survey, exercise self-efficacy scale, and insulin sensitivity check index	Low self-efficacy for resistance exercise was the most important predictor of patients' dropout	Moderate
Chen et al. [22]	Quasiexperiment; individuals with and without metabolic syndrome (MetS) (*n* = 110)	Three months of home-based exercise	Baseline and postintervention evaluated metabolic risk factors and exercise self-efficacy	Individuals without MetS had higher exercise self-efficacy than those with MetS; home-based exercise programs are beneficial for individuals at risk for diabetes	Weak

**Table 3:** Characteristics of studies using health promotion programs.

Author, reference	Study design/subjects	Intervention	Instruments/measurements	Outcomes/finding	Quality rating
Clark et al. [29]	Randomized controlled trial; adults with type 2 diabetes (*n* = 100)	Three-month lifestyle (diet and PA) self-management program (brief tailored) plus follow-up phone calls for one year	Diabetes self-management, self-efficacy for physical activity, and barriers to diabetes self-care were evaluated at baseline, postintervention, and after follow-up	↑ PA and ↓ dietary fat in the intervention group	Moderate
Toobert et al. [25]	Randomized controlled trial; postmenopausal type 2 diabetic women (*n* = 279)	Mediterranean lifestyle program (6-month intervention to construct group coach and 12- and 24-month follow-up); videotapes also used for home-based practice (one hour per day)	Lifestyle behaviors (i.e., physical activity and stress management) and psychosocial variables (e.g., social support, problem solving, self-efficacy, depression, and quality of life), at baseline and 6, 12, and 24 months	↑ Quality of life (stress management, healthy diet, exercise), psychosocial factors (self-efficacy, problem solving, supportive resources), after 12 and 24 months of problem-solving	Moderate
Baghianimoghadam et al. [26]	Randomized controlled trial; diabetic patients (*n* = 80)	Education programs on exercise-related behavioral changes based on the health belief model (2 sessions+ reminders in 3 months)	Questionnaire based on the health belief model, a checklist related to patients practice (before and three months after intervention)	Conducting patient walking training method → ↑ self-efficacy and knowledge about the disease management	Weak
Mladenovic et al. [27]	Qualitative substudy; type 2 diabetic patients (*n* = 13) completed healthy eating and active living for diabetes in primary care networks (HEALD) program	HEALD (primary care-based walking for 24 weeks) program	Semistructured interviews with purposefully selected HEALD completers six months after the program ended	↑ Exercise-related motivation and self-efficacy behaviors and postprogram contact with patients could improve those outcomes	Weak
Olson and McAuley [30]	Randomized controlled trial; older adults with type 2 diabetes titrated physical activity	Eight-week intervention included walking exercise and theory-based group workshops	Self-efficacy, self-regulation, and physical activity were assessed at baseline, postintervention, and a follow-up (6 months)	↑ Self-regulation/self-efficacy and physical activity at a two-month evaluation; ↓ beneficial effects at follow-up	Strong
Alharbi et al. [31]	Quasiexperiment secondary analysis of data collected from RCT; overweight adult (*n* = 134) with heart disease and diabetes	Healthy eating and exercise lifestyle program (group-based supervised structured exercise sessions, 4 months) plus telephone follow-up calls (4 months)	Exercise, self-efficacy for weight loss, and depressive symptoms were measured at baseline, postactive phase (4 months), and postmaintenance phase (12 months)	↑ Exercise adherence and weight loss; male gender, self-efficacy, time, and depressive symptoms are independent predictors for exercise duration	Moderate
Alharbi et al. [32]	Quasiexperiment secondary analysis of data collected from RCT; patients with coronary heart disease and/or diabetes mellitus (*n* = 134)	One year healthy eating and exercise lifestyle program	Self-efficacy for exercise survey at baseline, at 4 months, and at 12 months	Negative association between lack of motivation and exercise self-efficacy	Moderate
Anderson et al. [33]	Quasiexperiment; dyadic data from 117 married couples in which one partner was diagnosed with type 2 diabetes	—	Survey of two exercise items (diabetes self-care activities); seven-item self-efficacy subscale of the multidimensional diabetes questionnaire	Health stress of patients in the form of higher comorbidities number and specific stress of diabetes in both patient and spouse ↔ ↓ patient adherence to exercise ↔ diabetes self-efficacy and depressive symptoms	Moderate
Thoolen et al. [28]	Randomized clinical trial; type 2 diabetic (intervention, *n* = 78 or control, *n* = 102)	Self-management program (based on proactive coping and self-regulation theory in a five-step plan) lasted 12 weeks	Proactive coping, goal achievement, and self-efficacy were evaluated at baseline and postintervention	↑ PA and diet behavior, weight loss, coping, goal achievement, and self-efficacy	Moderate
Naik et al. [51]	Randomized pilot clinical trial; type 2 diabetic patients (*n* = 87)	Four sessions of clinician-led, patient-centered group education targeting type 2 diabetes self-management (medications, exercise, diet, home monitoring, etc.) for 3 months	HbA1c, self-efficacy scale, and specific knowledge and understanding scale at baseline, postintervention, and at the 1-year follow-up	↑ Glycemic control, self-management, and effect of time-by-treatment interaction might partially be mediated via the development of self-efficacy	Strong
Rosal et al. [34]	Randomized clinical trial; low-income Latin diabetic patients (*n* = 252)	Group-based intervention (12 weekly and 8 monthly sessions and targeted knowledge, attitudes, and self-management behaviors)	HbA1c, diet, physical activity, blood glucose self-monitoring, diabetes knowledge, and self-efficacy at baseline and at 4- and 12-month follow-up	↑ Self-efficacy and PA management at 4 months → ↓ HbA1C; ↓ statistical differences at 12 months, but ↑ patients' knowledge about diabetes	Moderate
Sperl-Hillen et al. [35]	Randomized clinical trial; adults with type 2 diabetes (*n* = 623)	Individualized education (IE, 3 sessions of 1-hour individual education once a month), or group education (GE, sessions of 2-hour group education) or control (UC) for 1 year plus 6.8 months and 12.8 months	HbA1c, general health status, problem areas in diabetes, diabetes self-efficacy, recommended food score, and physical activity evaluated at baseline, 3.8 months, and 6.8 months after randomization	↓ HbA1c in all groups; ↑ exercise score, self-efficacy, and HbA1c level of individual training group compared to group education and control group	Strong
Sperl-Hillen et al. [36]	Substudy of RCT; adults with type 2 diabetes (*n* = 623)	Sperl-Hillen et al., 2011 study intervention	Follow up evaluation of Sperl-Hillen et al. (2011) study at 12.8 months	Behavioral and psychological improvement in IE compared to GE and UC groups; however, no sustained improvement in HbA1c, nutrition, and PA scores	Strong
Tan et al. [37]	Randomized clinical trial; Malaysian diabetic patients (*n* = 151)	Face-to-face education program targeting self-efficacy on self-care skills for 12 weeks	HbA1c and revised diabetes self-care activities questionnaires (monthly and postintervention follow-up)	↑ Glycemic control, diet, medication adherence, and PA	Moderate
Van Dyck et al. [38]	Randomized clinical trial; adults with type 2 diabetes (*n* = 623)	Social-cognitive-based method (face-to-face education, telephone follow-ups) for 24 weeks	PA (pedometer, accelerometer, and the IPAQ) and change in psychosocial factors were measured at postintervention and 1-year follow-up	↑ Patients' self-efficacy and ↑ PA	Moderate
Jelsma et al. [39]	Randomized controlled study; women with gestational diabetes mellitus (*n* = 59)	Lifestyle-counselling sessions for 6 months (two face-to-face +5 telephone +5 text messages+4 mailed postcards)	Psychosocial determinants related to physical activity and diet were measured with a self-administrated questionnaire (at baseline and six months)	↑ Patients' self-efficacy and reduced barriers to active lifestyle such as lack of motivation and energy	Weak
van der Wulp et al. [41]	Randomized controlled trail; adults with recently diagnosed type 2 diabetes (*n* = 133)	Self-management coaching program (peer-led) on lifestyle changes (3 home visits targeting practical goals)	Self-efficacy, coping, physical activity, dietary habits, psychological well-being, depressive symptoms questionnaires at baseline and 3- and 6-month postintervention	↑ Scores of people with lower self-efficacy and psychological well-being index	Weak
Steed et al. [42]	Randomized controlled trail; patient with type 2 diabetes (*n* = 124)	Five weekly sessions of social cognitive (self-efficacy) and self-regulatory (illness beliefs) theory-based program	Revised summary of self-care diabetes activities (at baseline, one week, three months, and nine months)	↑ Self-efficacy for exercise immediately and three-month postintervention; essential role illness beliefs in the patients' quality of life, and self-efficacy in self-management behaviors	Weak
Campbell et al. [43]	Randomized controlled trial; adults with type 2 diabetes (*n* = 598)	Three-week intervention program (diabetes factsheets and a DVD comprising patient stories (narratives) of type 2 diabetes management with follow-up at 4 weeks and 6 months)	Diabetes management self-efficacy scale (A/E DMSES) and self-care activities (SDSCA) at baseline and 4 weeks	↑ Self-efficacy behaviors	Moderate
Gamboa et al. [44, 45]	Randomized controlled trial; adults with type 2 diabetes mellitus (*n* = 594)	Spanish Diabetes Self-Management Program (SDSMP)	HbA1c; Spanish diabetes self-efficacy scale at baseline and 6, 12, and 24 months after SDSMP	↑ Self-efficacy and self-management for controlling the disease; exercise self-efficacy changes were not significant	Moderate
Cioffi et al. [47]	Randomized controlled trial; overweight Asian Indian adults with prediabetes (*n* = 550)	Four-month diabetes prevention program on self-efficacy	Exercise-related self-efficacy was measured at baseline, core intervention completion (4 months), and annually until the end of follow-up (3 years or diabetes diagnosis)	↑ Self-efficacy at treatment completion, but this effect was not sustained over longer follow-up	Moderate
Moungngern et al. [46]	Randomized controlled trial; prediabetes subjects (*n* = 125)	Six-month group activities of health promotion protocol (Health Belief Model, the Self-Efficacy Theory)	Diet and exercise behavior questionnaire, the self-efficacy questionnaire	↑ Awareness, ↑ self-efficacy, and a realization of the benefits of health behavior modification	Moderate
King et al. [48]	Quasiexperiment on baseline data; diabetic patients (*n* = 463) with elevated BMI	—	Physical activity, adherence to diabetes, self-efficacy, and social-environmental variables were measured with different questionnaire and scale	↑ Psychosocial and social-environmental factors→ ↑ diabetes self-management; but independent association between self-efficacy factors with exercise	Weak
Dyck et al. [49]	Quasiexperiment; type 1 diabetes (T1D, *n* = 12) and diabetes care providers (DCP, *n* = 12)	Four weekly group sessions to learn about exercise physiology and experience different exercise types	Diabetes distress screening scale; physical activity and exercise Counselling survey in DCP	Intervention did not improve exercise self-efficacy of TID but improves DCP self-efficacy in providing exercise advice to patients	Weak
Powell et al. [50]	Quasiexperiment; diabetic patient counselor/educators (*n* = 119)	—	Evaluation of delivering diabetes self-management/support in diabetes educators	Challenging barriers were lack of enough time for delivering patient visits and inability to encourage patients for physical activity	Weak
Miller et al. [40]	Randomized controlled trial; prediabetic university employees (*n* = 68)	Sixteen-week group-based diabetes prevention program +3-month follow-up	Self-efficacy, behavioral self-regulation, and goal setting determinants were assessed at baseline, postintervention, and 3-month follow-up	Improvement in behavioral outcomes such as physical activity and diet self-efficacy and goal commitment in the intervention group	Strong

**Table 4:** Characteristics of studies using multimedia and education.

Author, reference	Study design/subjects	Intervention	Instruments/measurements	Outcomes/finding	Quality rating
Wangberg [55]	Two-group randomized trial; diabetes patients (*n* = 64) with highest self-efficacy (HSE) or lowest self-efficacy (LSE)	One month tailored Internet-based self-care management (diet, blood glucose or physical activity)	Diabetes self-care activities and competence scales	↑ Self-care behavior in both groups, but in HSE group was more than the LSE group	Weak
Sacco et al. [53]	Randomized control trial; diabetes patients (*n* = 62)	Telephone coaching intervention (brief and proactive) for 2 years	Glycemic control, diabetes self-care activities, self-efficacy, reinforcement for self-care activities, and awareness of self-care goals were measured	Awareness of self-care goals, self-efficacy, and reinforcement →↑ adherence to exercise and a healthy diet and ↓ medical complications and depression	Moderate
Lorig et al. [57]	Randomized control trial; diabetes patients (*n* = 761)	Online diabetes self-management program (with six trials and 18-month follow-up)	Health status, health behaviors, health care utilization, patient activation, and self-efficacy were measured	Improve HbA1C, exercise, patient activation, self-efficacy, and reinforcement or follow-up had no beneficial effect	Moderate
Wolever et al. [54]	Randomized clinical trial; patients with type 2 diabetes (*n* = 56)	Integrative health (IH) coaching (coaching was conducted by telephone for fourteen 30-minute sessions for six months)	Glycemic control, medication adherence, exercise frequency, patient engagement, and psychosocial variables were assessed	IH improved psychosocial outcomes (stress, exercise frequency, self-reported adherence, and self-efficacy)	Moderate
Khan et al. [60]	Randomized controlled trial; adults with type 2 diabetes (*n* = 129)	Computer multimedia diabetes education program (waiting room-administered, low-literacy)	Glycemic control, changes in behaviors, diabetes knowledge, self-efficacy, and medications prescribed were measured over 3 months	Multimedia-educated group had better adherence to oral medication but not for self-efficacy and other self-management behavior	Strong
Goodarzi et al. [58]	Randomized controlled trial; diabetic patients (*n* = 81)	Intervention group received 4 messages weekly about exercise, diet, and medication for 12 weeks	Patient's knowledge, attitude, practice, and self-efficacy were evaluated by questionaries	Smartphone communication increased the patients' self-efficacy in the intervention group	Moderate
Markowitz et al. [61]	Qualitative substudy diabetic patients completed and maintained physical activity after healthy eating and active living for diabetes program (*n* = 13)	Mobile-based healthy eating and active living for diabetes program	Interview questions focused on what participants liked or did not like about HEALD and their maintenance of physical activity six months after the program ended	This primary care-based walking program (24 weeks) was not effective to develop exercise-related motivation and self-efficacy behaviors	Weak
Block et al. [56]	Randomized controlled trial; prediabetes (*n* = 339)	Six-month online program (algorithm-driven) for prevention and improvement of diabetes	Five summary questions were asked on patients eating habits and one question on physical activity self-rated health status and self-efficacy	Improvement in achieving goals for self-efficacy and satisfaction, resulting in promoting physical activity behavior	Moderate
Lari et al. [52]	Randomized clinical trial study; adult with type 2 diabetes	Three-month education of diabetic patients using multimedia- (CD-) based health promotion model	Health promotion model questionnaires (self-efficacy; perceived benefits, barriers, and social support)	Intervention improved subjects' belief about PA and increase their adherence to exercise	Moderate
Lari et al. [59]	Randomized clinical trial study; adult with type 2 diabetes	Short message service- (SMS-) based model or multimedia counselling intervention	Health promotion model questionnaires (self-efficacy; perceived benefits, barriers, and social support)	Better effect of multimedia education on patients' self-efficacy and their belief about physical activity behavior than SMS	Moderate

## Data Availability

There is no raw data associated with this review article.
